# Community‐Based Physiotherapy Models and Neurological Trajectories in Infants at High Risk of Cerebral Palsy in Rural Tanzania: A Retrospective Routine‐Care Study

**DOI:** 10.1111/cch.70378

**Published:** 2026-11-08

**Authors:** Fernanda Cordeiro da Silva, Giovanna Dantas de Araujo, Natany Carla Lima da Silva, Giane Amorim Ribeiro‐Samora, Carolina Daniel de Lima‐Alvarez, Silvana Alves Pereira

**Affiliations:** ^1^ Acts of Life Morogoro Tanzania; ^2^ Federal University of Rio Grande do Norte Natal Brazil

**Keywords:** cerebral palsy, environmental enrichment, family involvement, HINE, physiotherapy, Tanzania

## Abstract

**Background:**

Cerebral palsy (CP) refers to a group of motor impairments resulting from nonprogressive brain malformations or injuries that occur early in development. Limited access to rehabilitation services in low‐ and middle‐income countries poses substantial challenges.

**Methods:**

This study aimed to describe the clinical and sociodemographic characteristics of children treated at a rural rehabilitation centre in Tanzania and to evaluate their neurological trajectories across three physiotherapy models. A retrospective longitudinal review (April to September 2025) analysed 126 medical records of children aged 3–24 months who underwent the Hammersmith Infant Neurological Examination (HINE) at the beginning of the intervention and after approximately 3 months of care. Participants were categorized into the hope intensive programme (HPP; 40 h/month), early intervention programme (EIP; 16 h/4 months) or conventional physiotherapy programme (CPP; 16 h/4 months).

**Results:**

Neurological function improved across all groups, with substantial raw neurological development gains reflected in the mean HINE scores, which increased from 41.6 to 55.3. Postintervention, the odds of infants remaining in the lowest HINE score range decreased significantly (OR: 0.07). The HPP group showed the fastest developmental progression. The study also supports the feasibility of using the Kiswahili‐translated HINE as an excellent low‐cost, responsive and context‐appropriate tool for longitudinal tracking and clinical monitoring.

**Conclusions:**

Structured neurorehabilitation, particularly intensive intervention, was associated with significant raw neurological progression in this low‐resource setting, highlighting the utility of the HINE for objective longitudinal surveillance.

## Summary

- The translated Kiswahili HINE is a highly responsive, low‐cost tool for longitudinal monitoring in resource‐limited settings lacking advanced neuroimaging.
- Intensive, structured community protocols combined with environmental enrichment significantly accelerate developmental progression in high‐risk infants.
- Integrating active family involvement, caregiver training and therapeutic play into daily routines drives raw neurological development gains.
- Centralized services and low health literacy in remote villages lead to late presentations, missing critical early neuroplasticity windows.
- Healthcare policies must prioritize funding for decentralized, community‐based rehabilitation to eliminate geographic barriers and reduce health inequities.

## 1 Introduction

Cerebral palsy (CP) is described as a set of motor disabilities resulting from malformations or nonprogressive lesions that develop in early childhood (Dan et al. 2025). Globally, the prevalence of CP is estimated at 2.11 per 1.000 live births, with lower rates in high‐income countries, at approximately 1.5 per 1.000, and higher rates in low‐ and middle‐income countries, reaching up to 3.14 per 1.000 (Brien et al. 2024). In certain Sub‐Saharan African regions, the prevalence may be even greater. Studies conducted in South Africa report rates around 10 cases per 1.000 live births (Duma et al. 2023). Specific epidemiological data for East African countries remain limited. However, United Nations Children's Fund (UNICEF 2021) estimates indicate that approximately 600 000 children and young people live with disabilities in Tanzania, many of whom present neuromotor conditions such as CP.

The high rates reflect persistent structural challenges in the region, such as late diagnosis, the lack of risk screening, shortage of specialized professionals and limited rehabilitation services, especially in rural areas. In Tanzania, one of the largest countries in East Africa and ranked 13th in terms of the continent's territorial area, there are only seven child rehabilitation centres, concentrated mostly in urban areas (National Bureau of Statistics (Tanzania) and Office of the Chief Government Statistician (Zanzibar) 2024; UNICEF 2021). This unequal distribution reinforces barriers to access for families living in remote and low‐income communities, increasing the risk of developmental delays and perpetuating the cycle of health inequity.

In these scenarios of structural limitations and inequalities in access, it is essential to discuss rehabilitation strategies that can be sustainably implemented in low‐ and middle‐income contexts (Kuper et al. 2022). In several parts of the world, innovative models of care for children with CP have been developed and evaluated, aiming to expand access and enhance the role of the family as a central agent in the therapeutic process (Karim et al. 2021; Maus et al. 2026). One example is the community‐based rehabilitation, widely employed in resource‐limited countries. In Bangladesh, Karim et al. (2021) demonstrated that community health worker‐led programmes, supported by logistical assistance such as free transportation, increased intervention reach and promoted caregiver training in daily disability management (Karim et al. 2021). Another emerging approach is telerehabilitation, which offers a feasible alternative in areas facing geographic barriers or shortages of specialized services. Maus et al. (2026) showed that, from the perspective of parents, this format favours continuity of care and reduces the costs associated with displacement to rehabilitation centres (Maus et al. 2026).

For families, geographic and service barriers translate into financial strain, disrupted caregiving routines and reduced ability to sustain follow‐up, making service models that integrate caregiver support and feasible access especially relevant (Kuper et al. 2022). The international literature, therefore, emphasizes that successful models of intervention for CP tended to incorporate family involvement, environmental enrichment and strategies of care contextualized to the sociocultural reality of each territory (Al‐Otaibi et al. 2024; Harniess et al. 2022). However, there is a significant gap in comparative studies on such approaches in East African countries, where access conditions, human resources and infrastructure are substantially different from the realities of high‐income countries.

In this context, the present study aimed to describe the clinical and sociodemographic characteristics of children receiving care in a rural Tanzanian rehabilitation centre and to evaluate their neurological trajectories over 3 months of physiotherapy across three distinct care models: the hope intensive programme, the early intervention programme and the conventional physical therapy programme. Through this, we aim to contribute to the global discussion on rehabilitation strategies that are effective and adaptable to low‐ and middle‐income contexts.

## 2 Methods

### 2.1 Study Setting

This study is part of a multicentre international project approved by a Central Research Ethics Committee in Brazil (CAAE: 75772223.3.1001.0253). It was conducted as a retrospective review (April to September 2025) of deidentified routine clinical records, from which direct personal identifiers (such as names and registration numbers) were removed, alongside HINE assessments collected during standard care at Acts of Life Tanzania in Morogoro District, Tanzania. No additional procedures were undertaken for research purposes, and there was no direct participant contact.

### 2.2 Study Design

This was a retrospective longitudinal study based on the review of deidentified clinical records and standardized neurological assessments using the Hammersmith Infant Neurological Examination (HINE). In total, 126 clinical records of children aged 3–24 months were reviewed, with all eligible cases and their respective repeated assessments included for analysis.

Participants for this study were part of a cohort of children receiving services through Acts of Life Tanzania's community‐based neurorehabilitation programmes in Morogoro. These programmes provide physiotherapy and caregiver support free of charge to families from across Tanzania, including rural villages and remote tribal communities, with the majority coming from low‐income households.

#### 2.2.1 Eligibility Criteria

Reflecting the specialized nature of the rehabilitation centre, the sample routinely comprised children with an established diagnosis of CP or those presenting a high risk for neurodevelopmental disabilities. Infants and young children were eligible if they had completed both a baseline and a 3‐month follow‐up HINE assessment during routine clinical care, performed between 3 and 24 months old. The corresponding clinical records were reviewed and categorized. Records with HINE forms incomplete beyond scoring feasibility, or with missing key demographic or perinatal variables, were not included.

#### 2.2.2 Study Groups

Participants were categorized into three clinical care pathways delivered as part of the centre's standard community‐based service delivery models, which were structured in alignment with international clinical practice guidelines for early intervention and motor learning in CP (Morgan et al. 2021; Novak et al. 2017):

1. House of hope intensive programme (HPP): children accommodated with their mothers at the *House of Hope residential family facility* for a continuous 4‐week stay, during which they participated in an intensive physiotherapy protocol consisting of 2 h of therapy per day, 5 days per week (Monday through Friday). Admission was reserved for families from remote villages, other regions or tribal communities outside the centre's municipality. Infants and young children between 6 and 24 months were included.
2. Early intervention programme (EIP): infants under 12 months of age who attended 1‐h physiotherapy sessions once a week, focusing on motor milestones training and therapeutic play interventions to promote early development.
3. Conventional physiotherapy programme (CPP): young children aged 13–24 months, who receive 1‐h conventional physiotherapy sessions once per week, following individualized rehabilitation goals.

#### 2.2.3 Description of Intervention Groups (as Documented in Clinical Records)

All infants and young children underwent a baseline evaluation on Day 1.

Grounded in dynamic systems theory and motor learning principles (Morgan et al. 2016, 2021), the intervention in the house of hope intensive programme emphasized infant‐initiated active practice, task‐specific functional training and environmental enrichment embedded in play. Daily sessions incorporated gentle preparatory mobilizations for trunk and pelvic dissociation, followed by active weight‐bearing exercises and step‐by‐step postural transitions (e.g., from floor postures to supported sitting, quadruped, kneeling and supported standing) tailored to the infant's motor capacity (Morgan et al. 2015, 2021). Therapists coached mothers daily to minimize passive handling, promote the child's autonomous motor exploration during play and reinforce responsive caregiver–child interaction (Dusing et al. 2018; Harniess et al. 2022).

For children in the conventional physiotherapy programme, the intervention applied the same evidence‐based principles of active motor learning and progressive postural training described above. Weekly sessions focused on individualized, goal‐directed functional activities, progressive weight‐bearing and therapeutic play to stimulate self‐initiated movement. In addition, mothers received coaching on responsive handling, correct positioning and strategies to foster active child engagement throughout daily routines at home.

For infants in the early intervention programme, sessions were tailored to the infant's corrected or chronological developmental stage, prioritizing early sensorimotor exploration, selective postural control and active transitions between developmental positions. Environmental enrichment, utilizing varied textures, visual stimuli and interactive toys, was incorporated to encourage self‐initiated reach, grasp and exploratory behaviours. Mothers were actively coached to support parent–infant reciprocity through touch, eye contact and shared play, integrating developmental facilitation into everyday parent–child interactions (Dusing et al. 2018; Harniess et al. 2022).

Although the therapeutic principles and goals were consistent across all three programmes, focusing on motor learning, postural transitions, therapeutic play and caregiver engagement, the main difference between them was the intensity and frequency of therapy. Children in the house of hope intensive programme received 40 h of intervention over 1 month (2 h per day, 5 days per week), whereas those in the conventional physiotherapy and early intervention programmes received 16 h of therapy across 4 months (1 h per week).

#### 2.2.4 Data Collection

Demographic variables (age at the moment of assessment, sex and region of origin), perinatal and neonatal history (gestational age, birthweight, delivery mode, documented perinatal asphyxia and neonatal complications), referral source and clinical comorbidities were extracted. Neurological data included the HINE global score, section subscores and asymmetry score. Gross motor function was categorized using the Gross Motor Function Classification System (GMFCS), as documented in the clinical records.

HINE assessments were strictly extracted from clinical records corresponding to two specific time points defined by the service's routine: a baseline evaluation completed on Day 1 of admission and a follow‐up reevaluation conducted at approximately 3 months of follow‐up for all participants, in accordance with the rehabilitation centre's standard clinical monitoring protocol.

### 2.3 Measures

#### 2.3.1 Gross Motor Function Classification System

Gross motor function severity was classified at baseline by trained physiotherapists using the five‐level GMFCS, Expanded & Revised (GMFCS‐E&R) (Palisano et al. 1997, 2008). The GMFCS is a standardized system that stratifies motor function in CP based on usual performance across home, community and care settings, incorporating the need for assistive technology and mobility devices; it shows robust content, construct, discriminative and predictive validity and has been widely adopted in research and clinical practice internationally (Palisano et al. 1997, 2008).

Age‐banded descriptors were applied per GMFCS‐E&R guidance (before 2 years; 2–4; 4–6; 6–12; and 12–18 years). Consistent with established literature indicating that GMFCS classification in infants can be less stable, classifications assigned under 2 years were considered provisional in routine clinical care, with formal clinical reclassification routinely scheduled at ≥ 2 years by the centre; however, only the provisional < 2 years baseline classifications were analysed in this study (Gorter et al. 2009; Palisano et al. 2008). The use of the GMFCS is well established in Sub‐Saharan Africa and comparable low‐resource contexts, supporting its feasibility for cohort characterization in resource‐limited settings (Donald et al. 2014; Fatudimu et al. 2014; Kakooza‐Mwesige et al. 2015, 2017). Given the clinical heterogeneity and the higher proportion of severe presentations in low‐ and middle‐income countries, incorporating the GMFCS provided a standardized functional anchor for between‐group baseline comparisons and contextualized HINE‐based risk categorization (Abate et al. 2024).

#### 2.3.2 Hammersmith Infant Neurological Examination (HINE)

Neurological assessment was performed using HINE, a standardized and validated tool designed to evaluate neurological function in infants aged 2–24 months. The HINE provides global and subscale scores assessing cranial nerves, posture, tone, movements and reflexes, as well as an asymmetry score. It is widely used in both clinical practice and research as an early marker of CP risk and as a prognostic tool for later motor outcomes.

Cut‐off values established in longitudinal cohorts have been shown to predict CP risk with high sensitivity and specificity, particularly when applied from 3 to 12 months of age. In the conventional HINE, cut‐off scores less than 40 at 3 months (sensitivity 85% and specificity 90%), less than 50 at 6 months (sensitivity 90% and specificity 85%), less than 56 at 9 months (sensitivity 95% and specificity 83%) and less than 63 at 12 months (sensitivity 98% and specificity 86%) demonstrated excellent predictive accuracy for CP (Haataja et al. 1999; Romeo et al. 2016, 2024).

The HINE has also been applied in low‐ and middle‐income country settings, including studies from Sub‐Saharan Africa that demonstrated its feasibility for early identification of infants at risk of CP and other neurodevelopmental disorders (Abate et al. 2024; Kakooza‐Mwesige et al. 2017; Martinello et al. 2017). In this study, the HINE was administered as part of routine clinical care by trained physiotherapists. The Kiswahili translation of the HINE and its accompanying guidance was produced by the research team, with official authorization from *Mac Keith Press* and are publicly available on the publisher's website (Hammersmith Neurological Examinations 2025). Although formal regional normative score validation remains an objective for future epidemiological research, this study provides the first clinical report on using the standardized Kiswahili HINE for longitudinal neurological monitoring in East Africa.

## 3 Procedures

### 3.1 Assessments

Data extraction and retrospective review of eligible archived clinical records were conducted by the research team between April and September 2025. All HINE and GMFCS evaluations had been previously administered and documented by trained physiotherapists during routine clinical care.

The HINE was administered to infants and young children aged 3–24 months, using the officially authorized Kiswahili translation and guidance developed by the Acts of Life team with permission from *Mac Keith Press*. To monitor neurological progression, the examination was applied at two distinct time points established by the centre's clinical routine: a baseline evaluation completed on Day 1 (baseline assessment) of enrolment and a standardized follow‐up reassessment conducted for all groups after approximately 3 months of care (follow‐up assessment). Extracted variables included the HINE global score, section subscores and asymmetry score. The behavioural section of the HINE was recorded to document the infant's behavioural state during the examination and to support interpretation of the neurological findings. Behavioural scores were analysed descriptively and were not included in the calculation of the HINE neurological optimality score.

Gross motor function was categorized using the GMFCS Expanded & Revised (GMFCS‐E&R) criteria applying the before 2 years age band, abstracted from clinical records at baseline. It is important to note that the group categorizations were based on the clinical records available; no experimental intervention was introduced as part of the study. The analysis was limited to a retrospective review of existing documentation.

### 3.2 Statistical Analysis

Descriptive analysis was performed, with quantitative variables expressed as mean (standard deviation) or median [first quartile and third quartile], and categorical variables presented as absolute and relative frequencies. Normal distribution of quantitative variables was assessed using the Shapiro–Wilk test. A bootstrap bias‐corrected and accelerated (BCa) method with 1000 replications was employed to obtain the respective 95% confidence intervals. Baseline sociodemographic and clinical characteristics were compared between groups using one‐way analysis of variance (one‐way ANOVA) with Tukey's post hoc test for multiple comparisons, where appropriate. Homogeneity of variances was tested using Levene's test, and Welch's correction was applied when necessary.

To evaluate total HINE scores and their respective subdomains, HINE scoring performance categories, differences between groups (HPP, EIP and CPP) and the interaction effects between groups and assessment time points (baseline vs. follow‐up), generalized linear mixed models (GLMMs) were used. For quantitative variables, a normal probability distribution with an identity link function was adopted. For categorical variables, a binomial probability distribution with a logit link function was applied, utilizing chronological age as a covariate. Participants were included in the models as random effects, whereas groups and assessment time points were treated as fixed effects. Post hoc analyses were conducted using Tukey's test for multiple comparisons. The level of significance was set at *p* < 0.05. All data were analysed using the Statistical Package for the Social Sciences (SPSS Version 23.0) and Jeffreys's Amazing Statistics Program (JASP Version 0.19.3).

## 4 Results

The study followed 126 children who performed two HINE assessments each. The participants were divided into three intervention models: 16 followed the protocol described in the HPP, 27 followed the EIP and 83 followed the CPP. Among the 126 participants, 81 were boys, and 45 were girls; the average age was 20.78 months in the HPP group, whereas in the EIP, the mean age was 9.22 months, and in the CPP, it was 18.57 months. The GMFCS was homogeneous among the groups (HPP: 4.56; EIP: 4.63; and CPP 4.39). The analysis of the clinical and sociodemographic characteristics of the three groups is described in Table 1.

**TABLE 1:** Participant characteristics.

Variables	Hope intensive programme	Early intervention programme	Conventional physiotherapy programme	*p* one‐way ANOVA
Gender				
Female	7/43.8	9/33.3	29/34.9	0.764
Male	9/56.3	18/66.7	54/65.1	
Mother's age	26.4 (6.0)	27.1 (5.3)	29.5 (6.3)	0.181
Gestational age	39.2 (1.7)	39.8 (2.2)	39.1 (2.5)	0.345
Chronological age	20.8 (11.6)*	9.2 (6.5)	18.6 (12.2)*	< 0.001
GMFCS	5.0 [4.7–5.0]	5.0 [4.0–5.0]	5.0 [4.0–5.0]	0.377

*Note:* Data expressed as mean (standard deviation), median [Quartiles 1–3] and absolute/relative frequencies.

* *p* < 0.05 compared to early intervention programme. This variable showed a nonnormal, highly skewed distribution (Shapiro–Wilk test, *p* < 0.05), which explains the large standard deviation relative to the mean. Nevertheless, all participants strictly met the age inclusion criterion (≤ 24 months). The format was adopted because the comparisons were performed using the bias‐corrected and accelerated (BCa) bootstrap method with 1000 resamples to estimate the corresponding 95% confidence intervals.

A significant difference was observed between baseline and follow‐up assessments for the total HINE score (*F*_*gl*(48)_ = 23.38; *p* < 0.001), with a mean increase of 13.50 points from baseline (41.56 ± 16.09) to follow‐up (55.03 ± 14.65) (Table 1), regardless of the group (*F*_*gl*(47)_ = 0.39; *p* = 0.677 to interaction).

When comparing HINE scores between baseline and follow‐up assessments, both the HPP and CPP demonstrated mean score increases across all subdomains. In the EIP group, cranial nerve scores improved at follow‐up (8.55 ± 4.93); however, the asymmetry score count also increased (5.20 ± 3.86) (Table 2).

**TABLE 2:** Longitudinal trajectories of HINE scores.

	HPP			EIP			CPP		GLMM *p* value		
	Baseline	Follow‐up	×	Baseline	Follow‐up	×	Baseline	Follow‐up	Baseline × follow‐up *p* value	Between groups *p* value	Interaction *p* value
Asymmetries	2.4 (1.6)	1.8 (1.7)		4.0 (2.8)	5.3 (3.9)		3.1 (2.2)	3.1 (2.5)	0.734	0.018*	0.486
Behaviour	13.1 (2.6)	14.5 (0.9)		12.9 (2.0)	11.0 (2.8)		13.2 (2.2)	13.4 (1.6)	0.800	0.119	0.175
Cranial Nerve Function	12.6 (2.9)	13.0 (2.4)		11.8 (2.4)	8.5 (4.9)		12.0 (3.4)	12.4 (2.7)	0.223	0.039**	0.057
Posture	10.0 (5.0)	11.1 (4.9)		9.7 (3.5)	10.4 (6.2)		10.8 (4.9)	12.0 (4.4)	0.369	0.533	0.984
Movements	3.3 (1.7)	3.6 (1.6)		2.6 (1.7)	3.6 (2.1)		3.3 (1.9)	3.3 (1.9)	0.307	0.848	0.588
Tone	16.9 (3.6)	19.6 (3.4)		17.1 (4.0)	15.6 (4.4)		17.3 (4.5)	18.8 (3.7)	0.313	0.270	0.247
Reflexes and reactions	6.8 (1.3)	9.30 (4.7)		6.77 (3.1)	5.87 (2.8)		8.3 (3.6)	8.8 (3.7)	0.438	0.146	0.356
Total	39.7 (12.0)	56.8 (14.8)		41.1 (13.9)	51.8 (16.9)		43.9 (15.3)	56.4 (13.1)	< 0.001***	0.564	0.677

*Note:* Data expressed as mean (standard deviation). Behavioural scores were analysed descriptively and were not included in the calculation of the HINE neurological optimality score.

Abbreviations: CPP, conventional physiotherapy programme; EIP, early intervention programme; GLMM, generalized linear mixed model; HPP, house of hope intensive programme.

* *p* = 0.018: difference from HPP compared to EIP, regardless of the time.

** *p* = 0.039: difference from EIP compared to HPP and CPP, regardless of the time.

*** *p* < 0.001: difference from baseline to follow‐up assessments, regardless of the group.

At baseline, the distribution of infants within the lowest HINE score categories was similar across all groups (*p* = 0.159), with 108 infants (85.7%) scoring below the standard age‐dependent optimality cut‐offs. Postintervention, the odds of infants remaining below these thresholds decreased significantly by 93.4% compared to baseline (OR: 0.07; 95% CI: 0.03–0.16; *p* < 0.001). This improvement occurred irrespective of the intervention type, as no significant interaction effect was observed (*p* = 0.171).

## 5 Discussion

This study demonstrates that children across all three models of care achieved meaningful raw neurological development gains, as evidenced by the significant increases in total HINE scores. At baseline, nearly 86% of infants scored below the standard age‐dependent optimality thresholds; however, by the 3‐month follow‐up, the vast majority demonstrated substantial metric progression within the scale. Rather than implying a statistical cure, these findings frame HINE as an excellent tool for clinical monitoring and longitudinal tracking, reinforcing the value of structured early intervention for mapping individual developmental trajectories in low‐resource settings.

These results align with international evidence showing that timely, activity‐based programmes can change developmental trajectories for high‐risk infants and reduce the severity of long‐term disability (Morgan et al. 2021; Novak et al. 2017). The largest and fastest gains were observed in the HPP. In the HPP model, children not only received more concentrated physiotherapy sessions but were also immersed in an enriched environment that provided constant opportunities for practice, stimulation and caregiver engagement.

These elements mirror the key principles of evidence‐based early programmes such as SPEEDI, GAME and COPCA, which emphasize goal‐directed activities embedded in daily life, enriched environments and parent coaching (Dusing et al. 2018; Morgan et al. 2015, 2016; Morgan et al. 2016). The experience from HPP indicates that intervention intensity, environmental context and caregiver involvement are not optional ‘add‐ons’ but critical drivers of progress, echoing the findings of systematic reviews of early interventions in infants with or at high risk of CP (Gladstone et al. 2017; Morgan et al. 2021).

Although overall raw HINE scores improved, some subdomains (cranial nerves, reflexes and asymmetries) showed less favourable change in the youngest group (EIP). These findings are best interpreted as the natural unmasking of neurological deficits over time specifically in infants under 1 year of age, rather than a lack of therapeutic benefit. The cranial nerve domain in particular warrants attention, as it encompasses not only swallowing but also vision (ocular motricity, visual pursuit) and hearing (orienting to sound). Subtle abnormalities in these functions often become clearer as infants mature and are clinically meaningful because early dysfunction in visual and auditory pathways is associated with later challenges in motor learning, communication and cognition (Fazzi et al. 2009; Song 2023). Reflex scores may have declined due to persistence of primitive reflexes beyond expected ages of integration, which is a well‐recognized risk marker for CP (Morgan et al. 2021; Romeo et al. 2024). Similarly, asymmetry may have increased as unilateral motor patterns became more consolidated, in line with evidence that the HINE asymmetry score is sensitive to hemiparetic presentations (Romeo et al. 2024).

A strength of this study is its real‐world African setting, where advanced diagnostics such as neuroimaging or specialized neurophysiology are rarely available. In this context, our findings suggest the practical utility of the HINE as a low‐cost tool for routine neurological surveillance in resource‐limited settings. Its administration requires minimal resources, can be completed quickly and has strong interrater reliability, even by trained nonphysician providers (Dubowitz et al. 1999). Importantly, this study used a locally translated version of the HINE (Kiswahili), marking one of the first documented applications of the tool in this region. This demonstrates that standardized neurological assessment can be adapted to low‐resource contexts and still generate meaningful, clinically actionable information (Gladstone et al. 2017; Romeo et al. 2016).

The present study has limitations. Because it was a retrospective chart review, allocation to programmes was based on convenience, resulting in unequal group sizes and baseline variations in characteristics such as chronological age, which introduced a potential selection bias. Importantly, these baseline age differences and nonrandom allocation preclude establishing a formal dose–response relationship between therapy hours and neurological outcomes; therefore, observed gains reflect the overall multicomponent care model rather than dose‐dependent causality alone. To mitigate the confounding effect of this baseline age asymmetry on clinical outcomes, chronological age was entered into our statistical models as a mandatory covariate. Additionally, the retrospective nature of the data limited our ability to closely control variations in individual family compliance to the scheduled follow‐up periods, and the inherent variability in the completeness of medical records remains a constraint. Furthermore, the absence of complementary diagnostic measures, such as the General Movements Assessment or neuroimaging, restricted baseline diagnostic precision (Morgan et al. 2021; Novak et al. 2017). Finally, because the centre selectively admits highly vulnerable families from remote regions, our sample is characterized by high baseline severity, which may limit the generalizability of the findings to milder neurodevelopmental cohorts. The predominance of higher GMFCS levels (IV–V) across all intervention groups reflects the real‐world clinical referral patterns in this Sub‐Saharan setting, where secondary specialized rehabilitation centres primarily receive infants with severe neurological sequelae and substantial functional limitations (Abate et al. 2024; Kakooza‐Mwesige et al. 2017). Additionally, because our study strictly included infants under 2 years of age, baseline GMFCS classifications were applied using the expanded descriptors for this age band, which are recognized as provisional (Gorter et al. 2009; Palisano et al. 2008).

Despite these constraints, the findings are compelling. Structured neurorehabilitation services, particularly when intensive and enriched, were associated with significant raw neurological development gains and substantial upward progression within HINE scoring brackets. The evidence suggests that increasing therapy dose and embedding stimulation into enriched daily routines can accelerate developmental progress, even in settings with limited resources. The use of the HINE, translated and applied locally in this study, proved to be both feasible and impactful, underscoring its value as a scalable solution for objective clinical monitoring and longitudinal tracking in low‐ and middle‐income countries (Gladstone et al. 2017; Romeo et al. 2016).

## Author Contributions

**Fernanda Cordeiro da Silva:** writing – review and editing, writing – original draft, investigation, methodology. **Giovanna Dantas de Araujo:** investigation, methodology, writing – review and editing, writing – original draft. **Natany Carla Lima da Silva:** validation, project administration. **Giane Amorim Ribeiro‐Samora:** data curation, formal analysis, validation, visualization, writing – review and editing, writing – original draft. **Carolina Daniel de Lima‐Alvarez:** writing – original draft, writing – review and editing, visualization, validation, project administration, supervision, resources. **Silvana Alves Pereira:** conceptualization, investigation, funding acquisition, writing – original draft, methodology, validation, visualization, writing – review and editing, formal analysis, project administration, data curation, supervision, resources.

## Funding

This study was supported by the Conselho Nacional de Desenvolvimento Científico e Tecnológico (441583/2023‐7) and the Coordenação de Aperfeiçoamento de Pessoal de Nível Superior (CODE001).

## Conflicts of Interest

The authors declare no conflicts of interest.

## Data Availability

The datasets generated and/or analysed during the current study are available from the corresponding author upon request.
